# Prevalence and Factors Associated with Atrial Fibrillation in Patients with Transient Ischemic Attack or Ischemic Stroke in Northern Vietnam

**DOI:** 10.3390/jcm12175516

**Published:** 2023-08-25

**Authors:** Phan Dinh Phong, Bui Nguyen Tung, Pham Manh Hung, Nguyen Ngoc Quang, Nguyen Thi Thu Hoai, Nguyen Viet Dung, Thanh N. Nguyen, Dao Viet Phuong, Mai Duy Ton

**Affiliations:** 1 Vietnam National Heart Institute, Bach Mai Hospital, 78 Giai Phong St, Phương Mai Ward, Dong Da District, Hanoi 10000, Vietnam; phong.vtm@gmail.com (P.D.P.); nguyentung1238@gmail.com (B.N.T.); hungmpham@gmail.com (P.M.H.); quangtm@gmail.com (N.N.Q.); hoainguyen1973@gmail.com (N.T.T.H.); vietdung.ump@vnu.edu.vn (N.V.D.); 2 Department of Cardiology, Hanoi Medical University, Hanoi 10000, Vietnam; daovietphuong85@gmail.com; 3 Department of Internal Medicine, University of Medicine and Pharmacy, Vietnam National University, Hanoi 10000, Vietnam; 4 Department of Neurology and Radiology, Boston Medical Center, Boston University Chobanian & Avedisian School of Medicine, Boston, MA 02118, USA; thanh.nguyen@bmc.org; 5 Department of Stroke and Cerebrovascular Disease, University of Medicine and Pharmacy, Vietnam National University, Hanoi 10000, Vietnam; 6 Stroke Center, Bach Mai Hospital, Giai Phong St., Phương Mai Ward, Dong Da District, Hanoi 10000, Vietnam

**Keywords:** atrial fibrillation, transient ischemic attack, ischemic stroke, prevalence, risk factors, Northern Vietnam

## Abstract

Background: The prevalence and risk factors of atrial fibrillation (AF) in patients with transient ischemic attack (TIA) or ischemic stroke in Northern Vietnam are not well understood. This study aimed to estimate the prevalence and identify factors associated with AF in this population. Methods: A cross-sectional study was conducted on 2038 consecutive patients with TIA or ischemic stroke admitted to Bach Mai Hospital. AF was diagnosed using an electrocardiogram or Holter monitor. Logistic regression analyses were performed to determine the association between AF and risk factors. Results: Among the patients, 18.1% (95% CI: 16.46 to 19.85) had AF. Older age, renal dysfunction, valvular heart disease (VHD), and low ejection fraction were significantly associated with AF. Advanced age (per 10 years) (adjusted OR, aOR 1.39; 95% CI, 1.23 to 1.57), estimated glomerular filtration ratio decrease (per 10 mL/min/1.73 m^2^) (aOR 1.12; 95% CI, 1.06 to 1.17), VHD (aOR 9.59; 95% CI, 7.10 to 12.95), and low ejection fraction (<50%) (aOR 2.61; 95% CI, 1.62 to 4.21) had notable odds ratios for AF. Conclusions: Atrial fibrillation is prevalent among patients with TIA or ischemic stroke in Northern Vietnam, surpassing rates in other Southeast Asian countries. Age, renal dysfunction, VHD, and low ejection fraction were significant risk factors for AF in this population.

## 1. Introduction

Cardioembolism is a common cause of ischemic stroke according to the TOAST classification. Atrial fibrillation is an independent factor for the development of cardiac thrombus and is associated with a five-fold increase in the risk of stroke [1].

Studies in Western countries in patients with TIA/ischemic stroke report an AF prevalence ranging between 20.4% and 33.4% [2]. Meanwhile, studies in Southeast Asia recorded an AF rate in a lower range from 5.8% to 23.4% [3,4,5,6,7]. Therefore, screening for AF plays an essential role in the primary and secondary prevention of stroke. To improve the effectiveness of secondary stroke prevention, it is important to identify the risk factors associated with AF. In 2014, a study conducted by Friberg L et al. in Sweden found a correlation between older age, female sex, VHD, HTN, and a history of stroke with AF [2].

In Vietnam, the incidence of stroke is on the rise, attributed to economic progress and shifts in lifestyle patterns. It was estimated that the number of newly diagnosed stroke cases reached 157,295 in 2021, considering a population estimate of 98.32 million individuals [8]. In addition to the issue of disease diagnosis, the identification of underlying causes of stroke, such as atrial fibrillation or atherosclerotic disease, carries an important significance in the management of stroke. Currently, only Tirschwell and Ton Thanh et al. (2012) at Da Nang Hospital reported the rate of AF in patients with ischemic stroke. With a total of 754 patients, including 328 patients with ischemic stroke, the rate of AF was recorded as 10%. However, the associated risk factors were not mentioned in the report [6].

Furthermore, owing to the geographical attributes characteristic of northern Vietnam, encompassing extensive mountainous and rural terrains inhabited by individuals afflicted by valvular heart disease, as well as densely populated urban and lowland areas accommodating a substantial populace, a noteworthy prevalence of metabolic disorders and chronic comorbidities is evident. As such, the incidence of atrial fibrillation exhibits regional disparities. Leveraging our central hospital’s strategic positioning and its capacity to cater to patients spanning these diverse localities, we undertook this study with the primary objective of evaluating the contemporary prevalence and delineating the risk factors associated with atrial fibrillation among patients presenting with ischemic stroke/TIA in the northern region of Vietnam.

## 2. Methods

### 2.1. Study Population

We prospectively studied consecutive TIA/ischemic stroke patients admitted to the Stroke Center (SC) and Viet Nam Heart Institute (VNHI), Bach Mai Hospital, from January 2021 to June 2022. Inclusion criteria were as follows: (1) age ≥ 18 years and (2) diagnosis of TIA/ischemic stroke was confirmed by the treating neurologist. All patients included in this study had either a 12-lead ECG or 24 h Holter monitor. Exclusion criteria were as follows: (1) patients with cerebral infarction due to trauma, infection, or metastatic malignant lesions and (2) being discharged or experiencing death before screening ECG by using either a 12-lead electrocardiogram (ECG) or a 24 h Holter monitor.

### 2.2. Data Collection

Baseline patient characteristics were collected and included age, sex, history of smoking, diabetes mellitus, hypertension, symptomatic peripheral vascular disease, hyperlipidemia, ischemic heart disease, and renal failure. Fundamental laboratory findings included creatinine, glucose, total cholesterol, LDL-C, HDL-C, triglyceride, Hba1c, electrocardiogram (ECG), and echocardiography. In this study, atrial fibrillation, encompassing all manifestations of intermittent, persistent, and permanent types, was diagnosed in accordance with the EHRA 2021 criteria, utilizing the expertise of physicians specialized in interpreting electrocardiograms [9]. The assessment of left ventricular ejection fraction (EF) employed either the Teichholz or Simpson methodologies, with the most conservative measurement being adopted as the definitive EF outcome [10]. Valvular heart disease refers to patients with moderate to severe mitral stenosis or prosthetic valves [11]. eGFR was calculated using the Cockcroft–Gault formula [12]. Data were collected from medical records and patient tests. Information from medical records was extracted using a predefined data collection form.

### 2.3. Statistical Analysis

All analyses were performed using STATA for Windows V.17.0 (Stata, College Station, TX, USA). Continuous variables are presented as mean (±SD) or median (interquartile range, IQR). Categorical variables are presented as frequency and percentage. Comparisons were assessed using chi-square (χ^2^) test or Fisher’s exact test for categorical variables and Student’s *t*-test or Mann–Whitney U test for continuous variables as appropriate. A two-tailed *p*-value < 0.05 was considered statistically significant.

Logistic regression analyses were applied to identify related factors for the prevalence of AF. Univariable logistic regression was performed on socio-demographic factors and other potential factors that may be related to AF, such as medical history, creatinine, valvular heart disease, and left ventricular EF. Only variables that had a *p*-value < 0.10 on univariable analysis were selected for multivariable analysis.

## 3. Results

### 3.1. Prevalence

A total of 2038 participants with a TIA or ischemic stroke were included over a period of 18 months. Among these participants, 369 patients (18.1% (95% CI: 16.46–19.85)) were diagnosed with AF. The study group consisted of 1324 males, accounting for 64.97% (95% CI 62.85–67.04) of the total population. The mean age of the participants was 65.7 ± 12.6 years. The prevalent comorbidities primarily consisted of hypertension (75.27% (95% CI 73.34–77.13)), dyslipidemia (40.48% (95% CI 38.34–42.65)), type 2 diabetes (22.8% (95% CI 20.49–24.15)), cerebrovascular disease (17.86% (95% CI 16.22–19.59)), and chronic kidney disease (10.7% (95% CI 9.39–12.12)). Baseline characteristics and vascular risk factors of the study population are shown in Table 1.

### 3.2. Risk Factor

Univariate logistic regression of potential risk factors for AF is presented in Table 2. In multivariate logistic regression, higher age (per 10 years) (OR 1.39; 95% CI: 1.23–1.57, *p* < 0.001), estimated glomerular filtration ratio (eGFR) decrease (per 10 mL/min/1.73 m^2^) (OR 1.12; 95% CI: 1.06–1.17, *p* = 0.001), VHD (OR 9.59; 95% CI: 7.10–12.95, *p* < 0.001), and low EF (<50%) (OR 2.61; 95% CI: 1.62–4.21) were associated with AF (Table 2).

## 4. Discussion

To our knowledge, this is the first study with a substantial number of patients describing the prevalence and risk associations of the presence of AF among Vietnamese stroke patients. The prevalence of AF in our study was found to be 18.4%, further affirming its role as one of the leading causes of cerebral infarction and transient ischemic attack (TIA) according to the TOAST classification [13]. This rate is relatively high when compared to other studies conducted in Vietnam and Southeast Asian countries such as Thailand, Malaysia, Singapore, and Indonesia [3,4,5,6,7]. However, the mean age and male predominance observed in our study were comparable to these aforementioned studies.

The reason behind this higher prevalence in our study can be attributed to the fact that it was conducted from a hospital-based study at Bach Mai Hospital. Among the 81 primary stroke treatment facilities in Vietnam, Bach Mai Hospital is the largest center in the Northern region, with a capacity to accommodate a minimum of 1000 patients annually [8]. Moreover, due to the typically more severe manifestation of ischemic stroke patients with atrial fibrillation (AF) during the acute phase, Bach Mai Hospital also assumes the role of a referral center for critically ill stroke patients from other medical facilities.

In reality, even with the utilization of a 24 h electrocardiogram, it remains challenging to screen all stroke patients for atrial fibrillation in clinical practice. Several studies have shown greater detection of AF with a prolonged period of monitoring [14]. Hence, although the rate of AF patients identified in our study was considerable, the actual prevalence may be even higher. This finding emphasizes the significance of recognizing the crucial role of registered nurses in stroke management within clinical practice, raising awareness about the importance of early detection and appropriate care for patients with AF.

When comparing our findings with studies conducted in Europe and America, such as those of Friberg 2014 and Alkhouli 2018 conducted in Sweden and the United States, we observed a similar trend regarding the association between AF and ischemic stroke/TIA [2,15]. Specifically, the rate of AF in patients with these conditions tended to increase with age, which aligns with the results reported in the aforementioned studies. In our study, advanced age was identified as a significant risk factor associated with an increased incidence of atrial fibrillation (AF) (OR 1.39, 95% CI: 1.23–1.57, *p* < 0.001). Moreover, age represents a high-risk factor for elevated stroke rates according to the CHA2DS2-VASc scale. Our study further demonstrates that age independently contributes to the occurrence of AF in patients with ischemic stroke. Notably, there is a dearth of studies conducted in Southeast Asia that address the combined impact of these risk factors, particularly age. However, when comparing our results with findings from studies conducted worldwide, we observed consistent outcomes. For instance, Friberg’s study categorized AF patients into distinct age groups (<60, 60–69, 70–79, 80–89, and over 90 years), revealing a progressively increasing association between age and the risk of developing AF with each successive 10-year interval [2]. These collective findings substantiate the prevailing understanding that age serves as an independent risk factor for AF development and related complications, such as stroke.

Interestingly, our study and those conducted in Europe (Friberg, 2014) and the United States (Fadar, 2016) present varying results regarding the gender distribution of AF patients [2,16]. In the European and American studies, a higher proportion of women with AF was observed, accounting for 29.9% and 19%, respectively, over the entire study population. In contrast, our study, as well as studies conducted in Southeast Asian countries, demonstrated a predominance of men not only among AF patients with ischemic stroke/transient ischemic attack (TIA) but also in the general population. According to the registry conducted by Ton et al., which involved over 10 centers in Vietnam, it was observed that males accounted for 60.7% of the ischemic stroke/TIA patients [17].

The presence of this male predominance with AF within the Southeast Asian populations was also observed in a study by Eitaro Kodani in 2012, conducted in both Korea and Japan [18]. This study included all hospitalized individuals receiving medical examination and treatment for various diseases and reported a higher prevalence of AF in men compared to women (2.7% vs. 2.3% in Korea and 2.7% vs. 2.3% in Japan). These findings suggest that the predominance of men with AF in our study, as well as in Southeast Asian countries, may be influenced by geographical and racial differences. The underlying reasons for these gender disparities in AF prevalence across different populations are likely multifactorial, involving a complex interplay of genetic, hormonal, and environmental factors.

Multiple studies have consistently demonstrated an association between declining renal function and an increased prevalence of AF [9,19]. Moreover, it has been established that patients with AF who also suffer from renal failure are at a heightened risk of ischemic stroke. These findings highlight the connection between chronic kidney disease and the occurrence of both AF and stroke. In our study, we discovered that every 10 mL/min/1.73 m^2^ decrease in glomerular filtration rate (eGFR) is a concurrent risk factor that amplifies the likelihood of AF in patients with ischemic stroke. This signifies the importance of monitoring renal function decline in patients with AF and ischemic stroke, as it contributes to the overall risk assessment. In Friberg’s study, however, renal failure was only identified as a positive risk factor in the univariate analysis and did not exhibit statistical significance in the multivariate regression (CI 0.97–1.18) [2]. Nonetheless, our study results highlight that the decline in renal function is indeed a significant risk factor that warrants monitoring in patients with AF and ischemic stroke.

Our study and previous research emphasize the significant associations between heart failure, valve disease, and the occurrence of atrial fibrillation. Heart failure is a significant risk factor included in the CHA2DS2-VASc score, encompassing both preserved ejection fraction (EF) (≥50%) and reduced or mildly reduced EF (<50%). In our study, due to the limited availability of NT-ProBNP testing, diagnosing heart failure with preserved EF was challenging. However, the presence of EF < 50% as a risk factor for atrial fibrillation further underscores the bidirectional relationship between these two conditions. Pathologically, both heart failure and atrial fibrillation contribute to an increased risk of thrombosis, while heart failure itself elevates the incidence of atrial fibrillation. These findings align with similar results from Friberg’s study, highlighting the association between heart failure and atrial fibrillation [2].

Valve disease is recognized as a classical factor contributing to the development of atrial fibrillation [20]. Patients with valvular heart disease often receive an atrial fibrillation diagnosis prior to their admission for ischemic stroke. Additionally, patients with valvular disease who are prescribed vitamin K antagonists (VKA) frequently encounter challenges in maintaining the prothrombin time–international normalized ratio (PT-INR) within the therapeutic range. It is worth noting that in Vietnam, acenocoumarol is predominantly used as a VKA medication, which adds to the difficulty of achieving optimal VKA dosing control. Consequently, patients may discontinue or avoid VKA treatment. Friberg’s study, despite dividing the valvular heart disease group into subgroups with or without mitral stenosis, consistently demonstrated a close relationship between valvular disease and the occurrence of mitral stenosis [2].

It’s noteworthy that risk factors implicated in ischemic stroke frequently coincide with those associated with AF, establishing a reciprocal relationship. This duality underscores the complexity of the clinical landscape, warranting a comprehensive approach beyond anticoagulant therapy alone. As highlighted by studies such as Wańkowicz et al. and Oladiran et al., the coexistence of these risk factors necessitates nuanced interventions that extend beyond anticoagulation to effectively safeguard patients with AF from the heightened risk of transient ischemic attacks (TIA) and stroke [21,22]. Hypertension stands as a well-established risk factor for AF within the general population and additionally serves as a risk factor for stroke among individuals with AF [23]. Interestingly, our study revealed a noteworthy finding: the cohort of stroke patients with hypertension exhibited a heightened likelihood of AF detection compared to their counterparts without hypertension. This finding contrasts with the observations in the study by Poh et al. [24]. Hence, further well-designed investigations are warranted to precisely ascertain the relationship between hypertension and AF within the stroke population.

The strengths of our study include the large number of consecutive patients with ischemic stroke studied in a tertiary hospital, screening with either a 12-lead ECG or 24 h Holter monitor in all patients, and prospective data collection minimizing recall bias and selection bias. With stroke care organized similarly throughout other regions of Vietnam, we aspire for the findings from this cohort to be applicable to the general stroke population. This extension of applicability is expected to yield a more comprehensive understanding of the burden imposed by atrial fibrillation across the entirety of Vietnam. Subsequently, these findings can be translated into practical applications by engaging with the media and healthcare professionals. This engagement seeks to enhance their awareness regarding the significance of screening and diagnosing atrial fibrillation, thereby contributing to the prevention of events like ischemic stroke/TIA. However, this study has some limitations. First, due to the burden of patient volume, some cases were rapidly discharged or transferred to other hospitals without fulfillment of blood testing. Second, Holter ECG was only indicated for limited use in patients with suspected atrial fibrillation and was not administered to all patients.

## 5. Conclusions

AF represents a significant contributing factor to TIA and ischemic stroke cases among patients in Northern Vietnam. In our study, the prevalence of AF was found to be lower compared to Western countries but higher compared to other Southeast Asian nations, which may be related to the duration of AF detection conducted. Noteworthy risk factors for AF included advanced age, renal dysfunction, valvular heart disease (VHD), and reduced ejection fraction (EF). These factors play a crucial role in increasing the likelihood of AF development in this population.

## Figures and Tables

**Table 1:** Baseline characteristics and comorbidities of included patients.

	Overall (*n* = 2038)		Atrial Fibrillation (AF)				*p*-Value
			Without AF (*n* = 1669)		With AF (*n* = 369)		
	$\overset{–}{x}$/*n*	SD/%	$\overset{–}{x}$/*n*	SD/%	$\overset{–}{x}$/*n*	SD/%	
Age (years)	65.7	12.6	64.8	12.6	69.7	12.1	<0.0001
Male gender, *n* (%)	1324	64.97	1124	67.35	200	54.20	<0.001
HTN, *n* (%)	1534	75.27	1298	77.77	236	63.96	<0.001
Hyperlipidemia, *n* (%)	825	40.48	662	39.66	163	44.17	0.110
Smoking, *n* (%)	51	2.50	43	2.58	8	2.17	0.854
Diabetes, *n* (%)	454	22.28	392	23.49	62	16.80	0.005
IHD, *n* (%)	99	4.86	71	4.25	28	7.59	0.007
PVD, *n* (%)	144	7.07	113	6.77	31	8.40	0.269
CKD (eGFR < 60 mL/min/1.73 m^2^), *n* (%)	218	10.70	156	9.35	62	16.80	<0.001
Gout, *n* (%)	67	3.29	59	3.54	8	2.17	0.257
Previous stroke, *n* (%)	364	17.86	285	17.08	79	21.41	0.049
Drugs							
Antiplatelet, *n* (%)	179	8.78	153	9.17	26	7.05	0.193
Antihypertension, *n* (%)	1152	56.53	967	57.94	185	50.14	0.006
Statin, *n* (%)	80	3.93	64	3.83	16	4.34	0.654
OAC, *n* (%)	124	6.08	34	2.10	89	24.12	<0.001
VHD, *n* (%)	307	15.06	121	7.25	186	50.41	<0.001
eGFR (ml/min/1.73 m^2^), $\overset{–}{x}$ ± SD	93.1	30.0	95.6	30.4	81.9	25.0	<0.0001
Glucose, median (IQR) (*n* = 1463)	7.1	5.9–8.9	7.1	5.8–9.0	7.1	6.2–8.5	0.4909
Cholesterol, median (IQR) (*n* = 1692)	4.7	4.0–5.4	4.8	4.1–5.5	4.4	3.7–5.2	<0.0001
Triglycerid, median (IQR) (*n* = 1687)	1.7	1.2–2.5	1.8	1.2–2.6	1.2	1.0–1.7	<0.0001
HDL, median (IQR) (*n* = 1666)	1.1	0.9–1.3	1.1	0.9–1.3	1.2	1.0–1.4	<0.0001
LDL, median (IQR) (*n* = 1685)	2.8	2.2–3.5	2.9	2.3–3.5	2.6	2.0–3.3	0.0003
EF ≤ 40%, *n* (%)	45	2.21	23	1.38	22	5.96	<0.001
EF 41–49%	74	3.63	30	1.80	44	11.92	

HTN: hypertension; IHD: ischemic heart disease; PVD: peripheral vascular disease; CKD: chronic kidney disease; eGFR: estimated glomerular filtration rate; OAC: oral anticoagulant; VHD: valvular heart disease; EF: ejection fraction; and IQR: interquartile range.

**Table 2:** Univariable and multivariable analyses: unadjusted and adjusted associations between risk factors and atrial fibrillation.

Total *n* = 2038	Univariate			Multivariate		
	Unadjusted OR	CI 95%	*p*	Adjusted OR	CI 95%	*p*-Value
Age (per 10 years)	**1.40**	**1.27–1.54**	**<0.001**	**1.39**	**1.23–1.57**	**<0.001**
Male sex	0.57	0.46–0.72	<0.001	0.79	0.60–1.05	0.106
Diabetes mellitus	0.66	0.49–0.88	0.005	0.81	0.57–1.15	0.236
Hypertension	0.51	0.40–0.65	<0.001	0.50	0.33–0.74	0.001
Coronary artery disease	1.85	1.18–2.91	0.008	1.12	0.64–1.95	0.694
Peripheral vascular disease	1.26	0.83–1.91	0.270	-	-	-
eGFR decrease (per 10 mL/min/1.73 m^2^)	**1.19**	**1.14–1.24**	**<0.001**	**1.12**	**1.06–1.17**	**<0.001**
Hyperlipidemia	1.20	0.96–1.51	0.111	-	-	-
Hypertension treatment	0.73	0.58–0.92	0.006	0.99	0.69–1.43	0.953
Valvular heart disease	**13.00**	**9.87–17.13**	**<0.001**	**9.59**	**7.10–12.95**	**<0.001**
EF < 50%	**6.64**	**4.53–9.73**	**<0.001**	**2.61**	**1.62–4.21**	**<0.001**
BMI (per 1 kg/m^2^)	0.94	0.89–0.98	0.009	1.04	0.98–1.09	0.221
Smoking	0.84	0.39–1.80	0.650	-	-	-

*p* < 0.10 and included in multivariable analysis. eGFR: estimated glomerular filtration rate; BMI: body mass index; EF: ejection fraction.

## Data Availability

Participant recordings have not been made publicly available to protect confidentiality. Further inquiries can be directed to the corresponding author.
